# Evaluation of China’s long-term care insurance policies

**DOI:** 10.3389/fpubh.2024.1252817

**Published:** 2024-03-28

**Authors:** Qiang Li, Yiwen Chen, Yongmei Zhang, Xue Liu

**Affiliations:** ^1^College of Economics and Management, Shandong Agricultural University, Tai’an, China; ^2^School of Economics and Management, Shandong Agricultural Engineering University, Jinan, China

**Keywords:** long-term care insurance, policy evaluation, fact-based assessment, value-based assessment, China

## Abstract

**Introduction:**

In response to the increasing demand for long-term care services for older people, the Chinese government has launched a pilot program for long-term care insurance (LTCI) since 2016. The objective of this study is to evaluate the performance and effectiveness of this program in China and provide recommendations for the future development and expansion of the LTCI system.

**Methods:**

We developed a comprehensive evaluation framework to assess these LTCI policies implemented in all 49 pilot cities in China.

**Results:**

Based on our evaluation, the average assessment score for the LTCI program across all pilot cities was 71.8 points, with scores ranging from 57.5 to 92.5 points in these cities. Furthermore, most of the pilot cities achieved higher scores in the fact-based assessment compared to the value-based assessment.

**Discussion:**

The results suggested that the overall pilot effect regarding LTCI was favorable, but there were significant regional disparities. Moreover, in most of pilot cities, current LTCI policies were designed to alleviate both the financial burden and the burden of caring for people with disabilities that families faced. However, some challenges still remained, such as the lack of community and home-based care services, the need to expand the coverage of insurance, and the importance of diversifying funding sources.

## 1 Introduction

**Figure 1:**
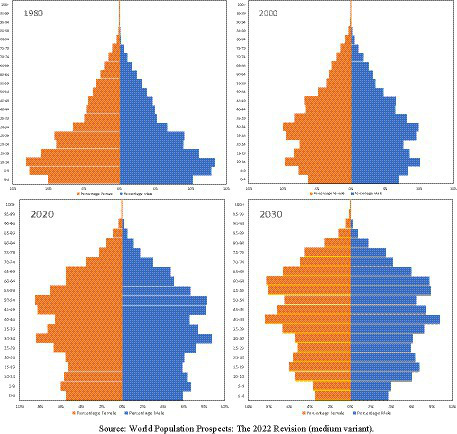
Population pyramids for China – 1980, 2000, 2020 and 2030. Source: World Population Prospects: The 2022 Revision (medium variant).

The advances in the medical and economic domains have led to an increase in life expectancy, resulting in a shift in the demographic distribution of population towards older age groups. Population aging poses a significant challenge not only to high-income countries, such as Japan, but also to many middle-income or even low-income countries (1). According to estimates from the World Health Organization, by the year 2050, two-thirds of the global population aged 60 and above will live in low- and middle-income countries (1). China is one of those countries that are experiencing the fastest demographic transition. The proportion of people aged 60 and above accounted for around 13.1 percent of total population in China in 2021, which is about 3 times higher than the figure in 1980. The population aged 80 years and above is projected to increase from 2.3 percent in 2021 to 10.3 percent in 2050 (2). However, the birth rate in China has been steadily declining over the years, reaching 6.8 births per thousand in 2022 (3). In comparison, the birth rate in the United States was 11 births per thousand, while in Japan it was 6.6 births per thousand in 2021 (2). These trends have resulted in a change in population age pyramid in China, characterized by a narrowing base and an expanding top (Figure 1). The growing population of older people directly contributes to a surge in the prevalence of older people with disabilities and the burden of caring for them (4, 5).

According to the Fourth Sample Survey of Living Conditions of the Elderly in Urban and Rural China, there are about 40.63 million older people with disabilities or semi-disabilities, representing 18.3 percent of the total older adults population in China (5). Family members are considered the primary caregivers for older people that have disabilities, however, the decline in fertility rates in China has led to smaller family sizes, thereby weakening their caregiving capacities (6, 7). To address the issue, the Chinese government initiated the first batch of pilot cities for LTCI in 15 cities in 2016. In addition, Shandong and Jilin Province were selected as national-level pilot provinces. These two provinces thus possess the autonomy to consider expanding the scope of pilot cities within their respective jurisdictions based on insights gained from the initial pilot outcomes. Subsequently, in 2020, the central government launched the second wave of long-term care insurance pilot projects. This expansion involves the selection of an additional 14 cities from provinces without existing pilot cities, in addition to 20 cities from Shandong and Jilin Province, bringing the total number of pilots to 49 cities.

LTCI system first emerged in European countries, with the Netherlands leading the way by implementing a universal public LTCI scheme in 1968 (8). Germany was the first country to implement public LTCI through social legislation, while Japan was the first country in Asia, followed by South Korea, to adopt public LTCI (8, 9). Many studies have reviewed and evaluated LTCI policies in these countries (10–12). There are also a body of literature focusing on the comparative and transnational studies of LTCI policies. Campbell et al. (13) examined the program goals, eligibility processes, scope, size, and sustainability in Germany and Japan to provide a model for the United States. Through comparing LTCI design in South Korea with Japan and Germany, Rhee et al. (14) suggested that middle-income countries should develop LTCI schemes early to have enough time to adapt the service delivery system before aging became a significant issue and substantial revenues were required.

Since the implementation of China’s pilot program for LTCI, a growing number of studies have endeavored to assessing the LTCI policies in China. Research on LTCI in China typically falls into two broad categories in terms of methodology: qualitative analysis and quantitative analysis. Literature concerning quantitative analysis primarily focused on examining the effects of LTCI on various economic and social aspects within one or some pilot cities by using micro-level data, such as CHARLS and CLHLS dataset.^1^ This included investigating the effects of LTCI on families’ medical expenses or consumption (15–20), the impact of LTCI on the use of medical services (21–24), the effects on older people’s health (24–26), and the spillover effects of the policies (27–29). In terms of qualitative analysis, it primarily addressed the question on how to improve LTCI program, with a specific emphasis on two main areas: Some studies reviewed the key characteristics of LTCI in other countries, such as in Germany, Japan or South Korea, to draw lessons and insights for China (9, 30). The other strand of literature focused on comparative analysis to compare core elements, such as funding sources and service provision, in some pilot cities (31–33). For example, Zheng et al. (32) constructed an evaluation framework based on 3 dimensions, the policies, management and supporting coordination to assess the performance and effectiveness of LTCI policies in three pilot cities, namely Chengdu, Qingdao, and Shijingshan district in Beijing.

Nevertheless, few studies dedicated to build an assessment framework that evaluates the policies of LTCI in all 49 pilot cities. Despite the National Healthcare Security Administration issued a guidance in 2020 (the Guidance 2020), it was only a policy document for reference. Each pilot local authority had the autonomy to formulate and tailor its own policies for LTCI. As highlighted in the research of Zhou and Dai (34), which investigated LTCI policies adopted in the first batch of 15 pilot cities, principles and characteristics of policies exhibited significant differences across pilot cities. Accordingly, the ultimate outcomes of LTCI program may differ resulting from these variations in policy formulation or measures taken by local governments.

The growing number of older people in China, together with the effects of the one-child policy that was enforced for more than three decades until it was relaxed in 2016, has created significant challenges and needs for long-term care services for older population (35). A comprehensive assessment of China’s LTCI is therefore essential, as it can provide insights into whether existing policies are adequately meeting the long-term care needs of the aging population. Additionally, China’s strategies for implementing LTCI may also serve as an example for other countries with similar demographic trends. However, as mentioned above, the existing literature has primarily focused on a selected number of pilot cities in China, overlooking the wide range of diversity and experiences across different regions (31–33, 36). This limited focus may not provide comprehensive insights into the key characteristics, challenges, and potential areas for improvement within China’s LTCI policies. Moreover, the lack of consistent assessment criteria makes it difficult to effectively compare policies implemented in different cities.

Therefore, in this paper, we provided an assessment of these LTCI policies implemented in each pilot city with the aim of providing insights and references to facilitate the establishment of a comprehensive and robust nationwide LTCI system. The study aims to fill the gap and make marginal contributions to the existing body of literature in several aspects. First, we developed a consistent framework for evaluating LTCI policies in all 49 pilot cities, from the dimensions of fact and value to introduce new ideas and insights into the study of LTCI policies. Second, these assessments allowed us to identify both positive aspects and challenges within the current LTCI policies in each pilot city. For example, our evaluation highlighted issues such as the lack of community and home-based care services, limited population coverage, and unsustainable financing modes during the current pilot phase. These can offer future direction not only for improving policies in each pilot city, but also for developing a consistent and effective national policy framework. In addition, this study can deepen the understanding of the variations in policy formulation among the pilot cities. This, in turn, may provide a novel explanatory mechanism for understanding the disparities in LTCI policy implementation outcomes among pilot cities in future research.

## 2 Materials and methods

### 2.1 Sampling and data collection

**Figure 2:**
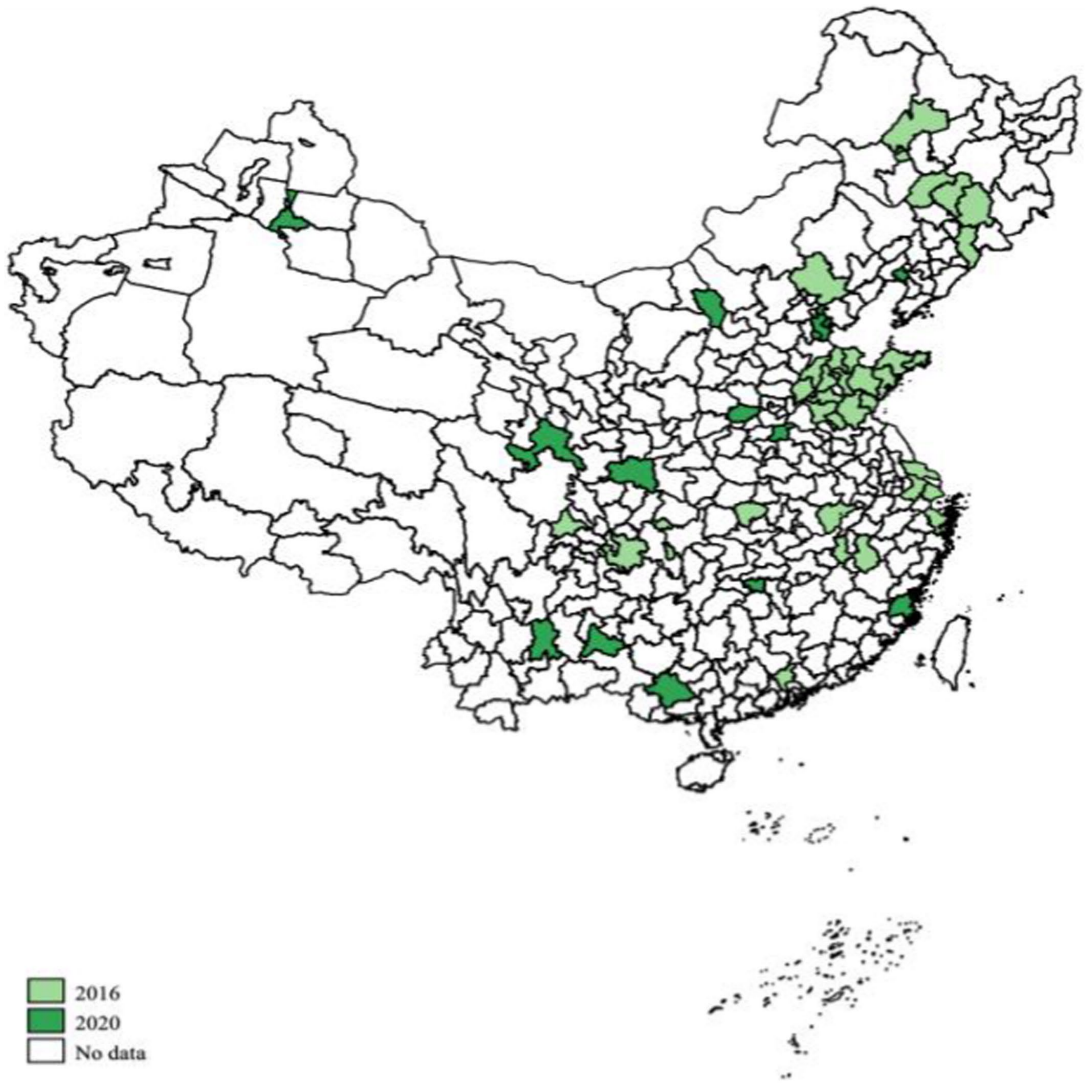
Geographical distribution of the pilot cities. Light green represents first batch of pilot cities that launched LTCI in 2016 and dark green represents second batch of pilot cities that launched LTCI in 2020. No data means the cities that are not pilot cities.

As of 2021, 49 cities have been included in the national pilot program for LTCI in China. Figure 2 displays the geographical distribution of the pilot cities. To carry out the evaluation, we conducted an extensive search of the official documents on LTCI issued by all the pilot authorities.^2^ These qualitative data were used as the primary data source for the indicators used in this paper.

In this study, we selected key indicators based on the Guidance issued by the central government in 2020 (hereafter referred to as “Guidance 2020”) as the criteria for building our evaluation framework.

Guidance 2020 refers to a set of guidelines issued by China’s National Healthcare Security Administration (37). They served as a strategic framework for implementing LTCI across various cities in China, drawing on the experience gained from the initial pilot program launched in 15 cities in 2016. The objective of the Guidance 2020 was to provide guidance and direction for policy practice in the pilot cities, focusing on eligibility, coverage, financing, and benefit levels. For example, it encouraged the local governments to adopt differentiated benefit policies based on levels of care and methods service delivery, while promoting the use of community-based and home-based care services. It also recommended coordinating LTCI with other policies and providing subsidies for people who are economically disadvantaged and have disabilities. These guidelines provided a valuable reference for developing a framework for evaluating LTCI policies in the pilot cities.

Drawing from the literature on Public Policy Analysis (38–41) and guided by the Guidance 2020 issued by China’s central government, we evaluated LTCI policies in each pilot city from the perspective of fact and value dimensions. As described by Guan (38) and Chen (39), fact-based evaluation in public policy analysis entails the use of criteria for evaluating and analyzing various factual aspects during policy implementation. Value-based evaluation refers to the criteria and the standards to be followed when assessing the value and implementation of a public policy. In contrast to quantitative analysis, which primarily measures “what is” or “what currently exists,” the evaluation based on fact and value criteria can allow us to go beyond and to explore “what ought to be” or what should be (40). It can provide insights into whether the policy formulation aligns with expected outcomes and social values. It can then shed light on how the policy is supposed to be implemented in theory by examining formal rules, structures, and procedures. In addition, it also allows us to identify inconsistencies, ambiguities or gaps in how the policy is articulated on the paper documents versus how it plays out in reality.

We selected six criteria: effectiveness, impact, responsiveness, equity, sustainability, and productivity. The first three—effectiveness, impact, and responsiveness—were part of the fact-based evaluation, which provided a direct description of policy effects. The remaining three—equity, sustainability, and productivity—fell into the category of value-based evaluation, which refers to the standards and criteria for making value judgments. Within these six criteria, we developed an evaluation framework consisting of 20 tertiary indicators, such as direct effects and indirect effects. A comprehensive overview regarding the description of each indicator is provided in Table 1.

**Table 1:** Description of indicators.

Tier 1 Dimension	Tier 2 Subdimensions	Tier 3 Indicators and the description of indicators
Fact-based assessment	Effectiveness	*Direct effects*: assessing whether LTCI can alleviate of caregiving burdens on families that have older people with disabilities
		*Indirect effects*: assessing whether LTCI can reduce the financial burdens on families that have older people with disabilities
		*Potential effects*: assessing whether LTCI can meet the healthcare needs of people with disabilities and alleviate the issue of “social hospitalization”
	Impact	*Impact on the long-term care services:* assessing whether LTCI can improve the socialized older adults service system
		*Impact on the long-term care service industry*: assessing whether LTCI policies can promote the development of long-term care institutions
		*Impact on the medical payment coverage*: assessing the payment coverage of LTCI
	Responsiveness	*Group-specific needs*: assessing whether the LTCI can meet the care needs of older adults with different levels of disability
		*Group-specific preferences*: assessing whether LTCI offers life-cycle services that cater to the preferences of different disability groups
		*Group-specific values*: assessing whether LTCI provides multi-level home-based and community-based care services.
Value-based assessment	Equity	*Equity in defining the target population*: assessing whether the coverage of LTCI is defined fairly
		*Equity in outcomes*: assessing whether the disability level criteria maximize the balance of treatment
		*Equity in rights*: assessing whether the rights to participate in LTCI are equal to all the residents.
		*Equity in opportunities*: assessing whether the eligibility criteria for accessing LTCI benefits are fair, ensuring that benefits are not subject to overly stringent conditions
	Sustainability	*Financial sustainability*: assessing whether the funding sources are diversified and whether the funding levels are dynamically adjusted based on the economic development of the cities
		*Sustainability of service delivery*: assessing whether the long-term care services are diversified and whether there is transferability between these services
		*Sustainability of administration operations*: assessing whether social forces are allowed to participate in administration and whether performance evaluation and incentive mechanisms are established
		*Sustainability of policy coherence*: assessing the degree of effective integration of LTCI with other social insurance programs and relevant functional departments
	Productivity	*Inclusiveness*: assessing whether the government plays a significant role in funding and subsidizing individual contributions for vulnerable groups
		*Adequacy*: assessing whether the funding level is in line with the level of economic and social development
		*Scientificity*: assessing the extent to which cities prioritize the development of a system platform and successfully implement intelligent operational processes and scientific service supervision within their LTCI

As previously mentioned, although the national guidelines exist, local authorities had the autonomy to stipulate their own policies for implementing LTCI. LTCI policies can vary across pilot cities in terms of the eligibility criteria, funding sources and covered services. The merit of the fact-based and value-based evaluation framework lies in its capacity to encompass both an assessment of the actual content of the policy and judgments about its social value. Focusing solely on one dimension could lead to inaccurate conclusion. Taking Shanghai as an example, looking solely at the fact-based dimension, the policies in Shanghai outperformed others in terms of its effectiveness, impact and responsiveness. However, when we considered the value-based dimension, particularly from the aspects of equity and sustainability, Shanghai’s LTCI had limitations. It covered only urban employees and urban residents, excluding rural residents, which resulted in incomplete coverage of older population. Furthermore, the policy was limited to people aged 60 or over, excluding those under 60 with disabilities and dementia. In addition, unlike in some cities where LTCI was funded through a combination of sources including social health insurance, government contributions, individual contributions, employer contributions, and other funding channels, the funding source for LTCI in Shanghai was singular, which may not be sustainable given the growing aging population. We provide an overview of the funding sources in pilot cities in Table 2, and further details are presented in Appendix 2. This indicated that LTCI in Shanghai needs to be improved in terms of equity and sustainability (value-based evaluation). Therefore, taking both dimensions into account is thus essential for a comprehensive and accurate policy evaluation.

**Table 2:** Funding sources of long-term care insurance (LTCI).

Funding sources	Cities
**Panel A: funding sources for urban employees**	
UEBMI	Shanghai, Suzhou
UEBMI + individual contributions	Chongqing, Jilin, Tonghua
UEBMI + individual contributions + government contributions	Chengde, Nantong, Anqing, Qingdao, Jingmen, Wulumuqi, Dezhou, Dongying
UEBMI + individual contributions + government contributions + public welfare lottery	Yantai, Jining, Weihai, Rizhao, Weifang, Taian, Heze, Linyi, Zibo, Zaozhuang
UEBMI + individual contributions + government contributions + employer contributions + public welfare lottery	Liaocheng
UEBMI + government contributions + public welfare lottery	Shihezi, Jinan
UEBMI + individual contributions + employer contributions	Shangrao
Employer contributions + individual contributions	Changchun, Qiqihaer, Ningbo, Guangzhou, Shijingshan, Tianjin, Panjin, Fuzhou, Kaifeng, Xiangtan, Nanning, Kunming, Songyuan, Meihekou, Hunchun,
Employer contributions + individual contributions + government contributions	Chengdu, Jincheng, Huhehaote, Hanzhong
Employer contributions + individual contributions + government contributions + public welfare lottery	Qianxinan, Binzhou
**Panel B: funding sources for urban non-working residents and rural residents**	
URRBMI	Shanghai, Suzhou
URRBMI + government subsidies	Yantai, Rizhao
URRBMI + individual contributions + government subsidies	Nantong, Shangrao, Jingmen, Huhehaote
URRBMI +government subsidies+ public welfare lottery	Weihai
Individual contributions + government subsidies	Changchun, Qingdao, Guangzhou, Chengdu, Shijingshan, Jinan, Dongying, Jilin, Songyuan, Tonghua, Meihekou
Individual contributions + government subsidies + public welfare lottery	Shihezi

Sources: data were collected through our compilation and summary of the long-term care insurance pilot programs. The information was extracted from official documents released by the local government of each pilot city. UEBMI and URRBMI are abbreviations for Urban Employee Basic Medical Insurance and Urban–Rural Resident Basic Medical Insurance, respectively.

### 2.2 Evaluation framework

#### 2.2.1 Scoring model

A four-category scale was employed to determine the scores for the evaluation indicators. Specifically, following the guidelines specified in the Guidance 2020, a score of 5 was assigned for full compliance with the guidelines of the Guidance 2020, 4 for substantial compliance, 2.5 for partial compliance, and 0 for non-compliance. Each tertiary indicator had a maximum score of 5, resulting in a total of 20 tertiary indicators and a cumulative score of 100. Detailed information on the scoring criteria can be found in the Appendix.

#### 2.2.2 Evaluation criteria

##### 2.2.2.1 The criteria for fact-based evaluation

###### 2.2.2.1.1 Effectiveness assessment

The assessment of effectiveness involved examining direct effects, indirect effects, and potential effects.

The direct effects of LTCI refer to the effects of LTCI on reducing the caregiving burden on families that have older people with disabilities. The long-term care services demanded by people with disabilities include not only basic daily care such as dietary and hygiene care, but also long-term medical care services such as nasogastric tube insertion and bladder irrigation. LTCI shall address both aspects to the ease caregiving burden on families. Long-term care services in the 49 pilot cities fell into three categories: those that focused primarily on daily living assistance, those that focused primarily on long-term medical care, and those that tried to maintain a balance between the two. The pilot cities that offered comprehensive long-term care services, including both long-term care for daily living and medical care, demonstrating full compliance with the Guidance 2020 and therefore receiving a score of 5 points. Cities that provided long-term care services predominantly in the form of either long-term daily living care or long-term medical care, demonstrating mostly compliant practices, and receiving a score of 4 points.^3^

Indirect effects refer to the effects of LTCI on reducing the financial burden on families with older people that have disabilities. A good LTCI benefit package can lead to decreased out-of-pocket expenses for people with disabilities, thereby alleviating their financial burden. The Guidance 2020 stated that the reimbursement rate for LTCI should be around 70%, which served as our classification criterion. Each pilot city was scored based on its reimbursement rate for institutional care. Five points were assigned to the cities where the reimbursement rate exceeded 75%. Cities with a rate between 65 and 75% received four points. Cities with a rate below 65% were assigned 2.5 points, indicating that the insurance in these cities only partially alleviated families’ financial burden.^4^

In terms of potential effects, the evaluation focused on whether LTCI in that city can effectively address the issue of “social hospitalization” among people with disabilities, thereby avoiding significant waste of medical resources. Social hospitalization represents the situation in which older people are admitted to long-term care hospitals for extended periods of time when their health care needs are relatively minor, such as simple care for minor illnesses. This can lead to unnecessary lengthy hospital stays, resulting in increased medical costs. Addressing the issue of “social hospitalization” requires attentions to providing multi-level long-term medical care system for patients with chronic conditions, such as cardiovascular diseases, such as cerebrovascular disorders and malignant tumors, and for people with severe disabilities. This enables a smooth transit from acute treatment to the long-term medical care, ultimately reducing the prolonged occupation of hospital beds by people with severe disabilities. A comprehensive LTCI should include three levels of care: specialized nursing care, institutional care, and home care. On the basis of this evaluation criterion, cities that provided all three levels of care services received 5 points. Cities provided two of the three types of care services received 4 points. Those that offered only one type of care services got 2.5 points.

###### 2.2.2.1.2 Impact assessment

LTCI explores the establishment of a long-term care service system that prioritizes home-based care with community and institutional support, further enriching and improving the long-term care services system. Moreover, by providing policy incentives and financial support, LTCI can stimulate the development of long-term care service institutions and increase employment opportunities in the care service sector, promoting the growth of the long-term care service industry. In addition, by building an integrated service platform that emphasizes home and community-based care, medical treatment, well-being and support, LTCI can effectively meet the needs of people with disabilities, avoid the high cost of long-term hospitalization, and thus improve the efficiency of the use of health insurance funds (37, 38, 42). Therefore, the assessment of impact in this study focused on 3 aspects: long-term care services, the long-term care services industry and medical payments coverage.

Long-term care services can be divided into two broad categories in terms of the forms of services: institutional care and home care. Institutional care further includes medical institution care and nursing institution care, while home care includes home visiting care and in-home care. Cities that offered all four types of services received 5 points as their policies fully in line with the Guidance 2020. Cities that provided both institutional care and home care, but where there was a partial lack of segmentation between two types of service, received 4 points. Cities that offered only one type of care services received 2.5 points.

The impact assessment of the long-term care service industry examined whether the pilot cities took active action in promoting the development of long-term care service industry, such as introducing talent training programs or issuing relevant policy directives to guarantee the implementation of LTCI. If the pilot cities introduced a comprehensive set of incentive policies to fully support the development of long-term care facilities and institutions and implemented talent training programs, they got 5 points. For example, Qingdao has set up comprehensive regulations to develop the long-term care service industry, including issuing specific policies to create incentive mechanisms and robust professional training programs. They thus obtained a 5-point rating. Cities that provided partial support for long-term care facilities and talent training programs, received 4 points. LTCI in some cities, such as Shangrao, has issued some policy documents to provide support for long-term care service facilities and professional trainings, but lacked relevant policy documents to support the creation of incentive mechanisms. They thus received 4 points. Cities that offered limited support to the long-term care services industry and lacked policies for relevant professional trainings, received 2.5 points.

The broader the coverage of medical payments, the clearer the functional positioning of LTCI and basic health insurance, the more obvious the solution to the problem of “social hospitalization.” Among the 49 pilot cities, they can be categorized into “limited,” “medium,” and “large” groups based on the coverage of LTCI payments. “Limited” indicated that medical payments covered only long-term care services, while “medium” represented the payments that included long-term care services, the use of equipment, and related consumables. “Large” indicated LTCI payments that covered not only long-term care services, the use of equipment and consumables, but also treatment, drugs and bed fees. Pilot cities with “large” coverage of medical payment received 5 points, cities with “medium” coverage obtained a score of 4 points, and cities with “limited” coverage were assigned 2.5 points.

###### 2.2.2.1.3 Responsiveness assessment

The responsiveness encompassed three dimensions: group-specific needs, group-specific preferences and group-specific values.

In terms of group-specific needs, as indicated by the theory of Maslow’s hierarchy of needs, different groups with different levels of disability have different needs (43). For instance, individuals with severe disabilities prioritize survival needs, while people with moderate disabilities emphasize security needs. Accordingly, the implementation of differentiated treatment and payment policies based on the levels of disability can effectively address group-specific needs. The assessment of group-specific needs examined whether each pilot city implemented a differentiated treatment policy. Within the 49 pilot cities, there were three categories of support based on the degrees of disability: firstly, support for severe disability; second, support for severe and some moderate disability; and thirdly, support for severe, moderate, and mild disability. Cities that effectively addressed the different needs of people with different levels of disability were awarded 5 points. Cities that provided support for groups that have severe and moderate disabilities received 4 points. Cities that only offered support for groups with severe disabilities and exhibited inadequate responsiveness to the needs of groups with mild or moderate disabilities received 2.5 points.

The assessment of group-specific preferences assessed whether LTCI offers life-cycle services that catered to the preferences of different groups. Given that the service preferences of different disability groups differ, the more comprehensive LTCI is, for example by adding hospice and end-of-life care services, the more it can respond to the service preferences of groups with disabilities. Pilot cities that provided life-cycle services, with a particular focus on providing hospice care and other services for terminal illness were awarded 5 points. Cities providing long-term care services across the life cycle, including medical care but excluding end-of-life care services for terminal illness, received 4 points. Cities that solely offered long-term life care services or incomplete long-term medical care services were assigned 2.5 points.

Group-specific values refer to the capacity of long-term care services to adapt to the “aging-in-place” values of specific groups. The extent to which home and community care services cater for different forms and content determines their responsiveness to the value of “aging in place” for people with disabilities. Therefore, the assessment of group-specific values examined whether each pilot city provided multi-level home-based and community-based care services. Providing care services in a familiar environment can help alleviate the anxiety experienced by people with disabilities, is consistent with the value of “aging in place” and better respects their feelings and dignity (44, 45). Pilot cities that provided community-based care and different types of home care, such as assistance with daily living for older people, home-based medical care and community-based home care, demonstrating a commitment to the value of “aging in place,” received 5 points. Cities that provided various forms of home-based care, both basic living care and medical care, but not community-based care, received a score of 4 points. Cities that provided only basic home-based care without community-based care received a score of 2.5 points.

##### 2.2.2.2 The criteria for value-based evaluation

Value-based evaluation covered the dimensions of equity, sustainability, and productivity, with a total of 11 indicators.

###### 2.2.2.2.1 Equity assessment

In the evaluation of equity, four key aspects were considered: the equity in defining the target population, equity in outcomes, equity in rights, and equity in opportunities.

From the perspective of equity in defining the target population, LTCI should cover all citizens, including both urban and rural residents, especially for rural people who are in relatively poor economic conditions. Moreover, inclusive LTCI should cover people with both physical and mental disabilities. Based on this criterion, if LTCI in the cities covered both urban employees and urban and rural residents, and extended coverage from the physically disabled to the mentally disabled, then the cities received 5 points; If the insurance in the cities covered urban employees and urban and rural residents, but the coverage was limited to people with physical disabilities, then the cities was awarded 4 points; For cities where only urban employees with physical disabilities were covered, they obtained 2.5 points.

Equity in outcomes represents that people with the same level of disability should receive similar treatment. The more comprehensive the factors considered in the disability level assessment criteria, the more accurately the level of disability is determined, enhancing the guarantee of treatment equity. In the 49 pilot cities, the disability level assessment criteria fell into three categories. The first category was based on the ability to perform activities of daily living. The second category included the ability to perform activities of daily living and the degree of illness. The third category assessed the level of disability based on the ability to perform activities of daily living, cognitive ability, and perceptual ability. Cities that assessed the level of disability based on the ability to perform activities of daily living, cognitive ability, and perceptual ability ensured equal treatment outcomes and received 5 points. Cities that used the ability to perform activities of daily living and the degree of illness as the criteria for determining disability were awarded 4 points. Cities that defined the level of disability based solely on the ability to perform daily living activities received 2.5 points.

Equity in rights means that all citizens can participate in LTCI without stringent conditions, such as age or identity restrictions. Therefore, the equity in rights assessed whether all individuals have the right to participate LTCI without restrictions based on age or identity status, such as their place of registration (hukou). For example, in Shanghai, participants in urban and rural medical insurance must be 60 years old or above. Cities with no restrictions on identity status and age for participation in LTCI received 5 points. Cities that had some restrictions on the age and identity status of urban and rural residents were awarded 4 points. Some cities where LTCI only covered urban employees and excluded urban and rural residents scored 2.5 points.

Equity in opportunities ensures that older people with disabilities have equal opportunities to access the benefits and payments without any additional conditions or limitations. The equity in opportunities examined whether the eligibility criteria for access to LTCI benefits were fair, ensuring that benefits were not subject to overly strict conditions. In the 49 pilot cities, there were two types of eligibility conditions for people that have disabilities to qualify for LTCI benefits: one was based solely on disability status, while the other imposed additional requirements such as enrollment duration, premium payments, and waiting periods. For instance, some cities required a one-year waiting period after premium payment for first-time participants or those who had interrupted their participation for more than six months. Cities that did not impose such conditions received a score of 5 points. Depending on the stringency of the eligibility requirements, cities were assigned scores of 4 points or 2.5 points.

###### 2.2.2.2.2 Sustainability assessment

Sustainability assessment is composed of four indicators: financial sustainability, sustainability of service delivery, sustainability of administrative operations, and sustainability of policy coherence.

Financial sustainability refers to gradually reducing reliance on existing social health insurance and establishing diverse funding channels that align with the level of economic and social development.^5^ The financial sustainability of LTCI was assessed by examining the presence of multiple funding sources and the ability to adjust financing criteria dynamically. Diversifying funding channels and implementing flexible financing criteria and diversify funding sources to ensure long-term sustainability of a LTCI system. Funding standards can be fixed, based on a fixed ratio, or a combination of both, with the latter two allowing for dynamic adjustments based on the economic development of the cities (1, 46). According to the guideline, cities that implemented LTCI policies with multiple funding channels, dynamically adjustable standards, and a relatively low proportion of medical insurance funding received 5 points. Cities with diversified funding channels, dynamically adjustable standards, but a high proportion of medical insurance funding received 4 points. Cities that had dynamically adjustable funding levels but lack diversification in funding sources received 2.5 points.

Service delivery sustainability ensures the integration of home-based, community-based, and institution-based care services to meet the different needs of people with different levels of disability. Given that the disability status of various groups with different levels of disability changes dynamically, the required type and content of care services also vary. Therefore, it is crucial to establish a transfer mechanism between different care services to minimize harm to people with disabilities and prevent the waste of resources resulting from inadequate coordination among care services. Therefore, cities are awarded 5 points if they offered a diverse range of services, such as hospital care, nursing home care, full-day home care, and part-day home care. Additionally, these cities should allow for the transfer of services between the aforementioned types through a registration process to accommodate changes in the disability condition and degree. Cities that provided multiple types of services but lack sufficient flexibility in transferring between them received 4 points. Conversely, cities that solely offered a single type of service, such as institutional care, received 2.5 points.

The sustainability of administrative operation refers to actively involving social forces in the administration of LTCI, accelerating the development of system platforms and the innovative application of technologies and continuously improving the operational service capacity and efficiency. Sustainability of administrative operations was assessed by examining whether social forces were allowed to participate in administration and whether performance evaluation and incentive mechanisms were established. These operations can help clarify the government’s functions, leverage the professional capabilities of commercial insurance companies, alleviate staff shortages and operational pressures, thereby enhancing the sustainability of administrative operations. Cities received 5 points if they actively engaged commercial insurance companies in administrative operations and established comprehensive performance evaluation and incentive mechanisms to improve the efficiency of management and services. Cities received 4 points if they involved commercial insurance companies in administrative operations but lack a comprehensive assessment mechanism. Cities got 2.5 points if they did not involve social forces in operations and exhibited deficiencies in their performance evaluation framework.

Sustainability of policy coherence requires the need for effective coordination and integration between LTCI and other relevant social security systems. Sustainability of policy coherence assessed the effective integration of LTCI with other social insurance programs and relevant functional departments. Cities that demonstrated excellent policy coherence in LTCI received 5 points, indicating strong connections with other social insurance programs like health insurance and work injury insurance. These cities exhibited clear scope of reimbursement and robust coordination and collaboration among relevant departments. Cities with good policy coherence received 4 points. However, cities with LTCI policies that demonstrated a significant need for improvement in departmental coordination and exhibited low efficiency received 2.5 points.

###### 2.2.2.2.3 Productivity assessment

The evaluation of productivity was carried out from three aspects: inclusiveness, adequacy, and scientificity.

Inclusiveness involves the government’s role as a funding entity and its provision of a safety net for particularly vulnerable groups, ensuring their access and participation in the program. The assessment of inclusiveness considered whether the govern significantly financed and subsidized individual contributions for vulnerable groups, such as low-income families. Cities that had a local authority as the main funding body and provided full subsidies for the individual contributions of vulnerable groups were assigned to 5 points. Cities where the local government was a major funding body and partially subsidized the individual contributions of vulnerable groups received 4 points. Cities where the government is one of the main funding bodies or provides subsidies solely for the individual contributions of vulnerable groups were assigned 2.5 points.

The assessment of adequacy focuses on aligning funding levels of LTCI with prevailing economic and social development, specifically in terms of funding levels that correspond to the pace of economic growth. It is important to ensure that funding levels correspond to the pace of economic growth in order to maintain adequacy. The level of economic development was proxied by the *per capita* GDP, while the funding level was represented by the total amount of funding raised in each pilot city. The average value of funding amount and *per capita* GDP were used as the classification standard. Cities received 5 points if the funding level of LTCI fully aligned with the level of economic development. If the funding level partially corresponded to the economic development level, cities were awarded 4 points. Cities that had a funding level slightly below their economic development level received 2.5 points. Cities with funding level far lagged behind their economic development level received a score of 0.

Scientificity emphasizes the need for a comprehensive platform for LTCI that enables intelligent and scientifically-driven management and operations. Scientificity evaluated the extent to which cities prioritize the development of a system platform and successfully implement intelligent operational processes and scientific service supervision within their LTCI. Cities with excellent scientificity in LTCI received a score of 5 points, indicating that they actively promoted the construction of a robust information system platform and comprehensive database containing detailed information about people that have disabilities. In addition, these cities had well-established scientific service supervision practices to ensure the delivery of high-quality and efficient LTCI services. Cities with good scientificity received a score of 4 points, denoting their commendable efforts in this area. For cities with a fair scientificity, a score of 2.5 points was assigned, reflecting their moderate progress on that respect.

#### 2.2.3 Weights

In this study, we used the equal weighting, in other words, we assigned equal weights to each indicator in order to emphasize the equal importance and independent existence of each indicator in the overall assessment.

#### 2.2.4 Index calculation

Since each subdimension consists of several indicators, all the indicators under a certain subdimension were aggregated according to Eq. 1.


(1) $$f_{i}=\frac{1}{n_{i}}\overset{n_{i}}{\underset{j=1}{\sum }}r_{ij}$$

where $f_{i}$ the index score of subdimension i , and $r_{ij}$ represents the score of indicator j in sub-dimension i . $n_{i}$ is the number of indicators in subdimension i .

Finally, the fact-based evaluation score and the value-based evaluation score were calculated as the average of the sum of scores for their respective subdimensions.

## 3 Results

In this section, we present the results that are obtained based on our evaluation framework.

The summary of the assessment scores is presented in Table 3. The assessment scores of the pilot cities ranged from 57.5 to 92.5 points, with an average score of 71.83 points, suggesting that the implementation of LTCI in China’s pilot cities was generally effective and successful, but there were large variations across the cities. The 49 pilot cities can be categorized into three groups based on their scores. Among them, there were 9 pilot cities with excellent performance (score ≥ 80), accounting for 18.4% of the total number of pilots, including Qingdao, Jingmen, Shanghai, Nantong, Chengdu, Suzhou, Guangzhou, Hohhot and Jinan. There were 23 pilot areas with good performance (70 ≤ score < 80), accounting for 46.9% of the total number of pilots. 17 pilot cities were assessed as fair performance (score < 70), accounting for 34.7% of the total number of pilots. The total score of fact-based assessment was 45 points, with an average value of 33.3 points (equivalent to 74 points in the 100-point grading scale); the total score of value-based assessment was 55 points, with an average value of 38.5 points (equivalent to 70 points in 100-point grading scale). Among them, 69.4% of the pilot cities obtained a higher score in the fact-based assessment compared to the value-based assessment.

**Table 3:** Summary of the mean values of assessment scores of indicators.

Indicators	Average score (points)	Indicators	Average score (points)
Effectiveness: 12.20		Equity in defining the target population	3.24
Direct effects	4.16	Equity in outcomes	3.17
Indirect effects	4.15	Equity in rights	3.46
Potential effects	3.86	Equity in opportunities	4.63
Impact: 11.80		Sustainability: 15.20	
Impact on long-term care services	4.07	Financial sustainability	3.23
Impact on the medical payment coverage	3.77	Sustainability of service delivery	3.95
Impact on the long-term care service industry	3.96	Sustainability of administration operations	4.04
Responsiveness: 9.40		Institutional cohesion sustainability	4.00
Group-specific needs	3.17	Productivity: 8.80	
Group-specific preferences	3.02	Inclusiveness	2.02
Group-specific values	3.18	Adequacy	3.24
*Equity:14.50*		Scientificity	3.45
Average score (points)		3.59	

### 3.1 The assessment scores based on fact-based evaluation

Qingdao and Shanghai were awarded the highest scores (43 points) in terms of fact-based evaluation. 26 out of the 49 pilot cities were scored above average, representing 53.1% of the total. From the perspective of the subdimensions of the fact, it showed that the ranking of the indicators’ scores was effectiveness (12.2 points) > impact (11.8 points) > responsiveness (9.4 points). The detailed information about the scores of fact-based assessments is displayed Appendix 3.

#### 3.1.1 Effectiveness

The average scores of the effectiveness indicators in the 49 pilot cities were ranked as direct effects (4.16 points) > indirect effects (4.15 points) > potential effects (3.86 points), all of which were greater than the overall average, indicating that the indicators under the effect dimension have achieved favorable outcomes The high scores of direct and indirect effects suggested that the LTCI policies implemented in the pilot cities were able to alleviate the burdens of caregiving and financial burdens of older people that have disabilities. Potential effects were also favorable, albeit with comparatively lower effectiveness compared to the other two effects. This suggested that efforts to mitigate “social hospitalization” have been made to some extent, yet further improvements are necessary.

#### 3.1.2 Impact

The average values of the scores of indicators regarding the dimension of impact were ranked as the long-term care services (4.07 points) > long-term care services industry (3.96 points) > medical payment coverage (3.77 points), all three of which were greater than the overall mean value, indicating that the implementation of LTCI had a positive impact on the above three indicators. Specifically, it improved the socialized older adults service system for people with disabilities, promoted the construction of long-term care service institutions and talent teams, and improved the efficiency of the use of medical insurance funds.

#### 3.1.3 Responsiveness

The average score of responsiveness indicators in 49 pilot cities was ranked as group-specific values (3.18 points) > group-specific needs (3.17 points) > group-specific preferences (3.02 points), all of which were less than the overall average, suggesting that the indicators under the responsiveness dimension performed not well.

We found that 28 out of the 49 pilot cities, LTCI programs were limited to offering care or treatment only to people with severe disabilities, excluding those with moderate or mild disabilities. Furthermore, only 9 pilot cities included hospice and end-of-life care services in their LTCI programs. In terms of group-specific values, only 4 cities, namely Shanghai, Suzhou, Jinan, and Zibo, provided multi-level community-based and home-based care service options. For instance, Suzhou offered home basic living care services, home-based medical care services, and community-based care services to meet people’s value of “aging in place.” Consequently, the overall impact of the pilot programs was not notably significant, resulting in inadequate fulfillment of the needs, preferences, and values of these specific groups.

### 3.2 The assessment scores based on value-based evaluation

As shown in Table 3, in terms of value-based evaluation, the total assessment score was 55 points. The assessment scores of 49 pilot cities were between 32–49.5 points, with an average of 38.5 points. Qingdao got the highest score (49.5 points) while Qiqihar obtained the lowest score is (32 points). In 49 pilot cities, the assessment scores of 24 pilot cities, representing 49.0% of the total number of pilots, were greater than or equal to the average. With respect to subdimensions of the value, the ranking of the effect scores was sustainability (15.2 points) > equity (14.5 points) > productivity (8.8 points).

#### 3.2.1 Equity

In terms of equity, the scores of indicators were ranked in the following order: the equity of opportunities (4.63 points) > the equity of rights (3.46 points) > the equity of defining the target population (3.24 points) > the equity of outcomes (3.17 points). The average score for equity of opportunities exceeded the overall average, while the remaining three indicators fell below the overall average. This indicates that LTCI did not perform well in terms of equity. With the exception of the high score for equity of opportunities, the scores for the other three indicators were notably low. In other words, the majority of pilot cities imposed certain restrictions based on identity status or age for individuals seeking to participate in LTCI. Additionally, LTCI may not cover all urban and rural residents, leaving certain groups, such as those with dementia, without sufficient protection. Furthermore, the fragmentation of assessment criteria posed a significant challenge, hindering the mutual recognition of disability status and impeding equal access to LTCI benefits.

#### 3.2.2 Sustainability

The average score of indicators with respect to sustainability was ranked as follows: sustainability of administration operation (4.04 points) > sustainability of policy coherence > sustainability of service delivery (3.95 points) > financial sustainability (3.23 points). The first three index all surpassed the overall average, indicating notable achievements in terms of operation, policy coherence, and service delivery. However, the score for financial sustainability fell below the overall average, suggesting a lack of independence in LTCI financing and inadequate flexibility in adjusting financing standards. Therefore, further improvement of the financing mechanism is necessary.

#### 3.2.3 Productivity

The mean values of indicators regarding the productivity were ranked as follows: scientificity (3.45 points) > adequacy (3.24 points) > inclusiveness (2.02 points), all three of which were below the total average, suggesting that the current policies implemented in the pilots do not perform well in terms of productivity. The scores for scientificity and adequacy indicator just slightly fell below the mean value, indicating some progress in the development of the LTCI system’s information platform and innovative applications. However, there was still room for improvement in terms of information sharing and integration with care service providers and other platforms. The score for inclusiveness indicator was relatively low, highlighting the limited benefits that vulnerable groups, such as individuals with disabilities and those with low incomes, can derive from economic and social development. Consequently, there is a need to strengthen the government’s role in subsidizing and supporting these individuals.

## 4 Discussions

With a growing aging population, the provision of long-term care for people that have disabilities has raised concerns for both policymakers and academic researchers. In 2016, China initiated LTCI by selecting 15 cities and 2 provinces as pilot sites. In 2020, the central government issued the “Guidance 2020,” extending the pilot cities to a total number of 49. As each pilot local authority has the autonomy to implement different policies and measures for LTCI, there may exist variations in LTCI across cities. Therefore, in this study, we assessed the LTCI policies implemented in each pilot city with the aim to provide insights and references for the establishment of an efficient and effective long-term care system for the seniors.

We developed an evaluation framework by selecting crucial indicators and setting specific evaluation criteria for each of them. After conducting an assessment of the relevant policies from the perspectives of facts and values, we observed that the evaluation scores of LTCI in 49 pilot cities ranged from 57.5 to 92.5 points. The average score across all cities was 71.8 points. Notably, 9 pilot cities demonstrated outstanding performance in terms of their LTCI system, accounting for 18.4% of the total number of pilot cities. These cities include Qingdao, Jingmen, Shanghai, Nantong, Chengdu, Suzhou, Guangzhou, Hohhot and Jinan. However, 17 cities exhibited only fair performance, making up 34.7% of the total number of pilot cities. This indicated that the overall pilot effect was favorable, but there were significant region disparities in LTCI among cities. The results are consistent with the existing literature. Zhou and Dai (34) examined the long-term care insurance systems across the 15 cities and found that there were many inconsistencies across these areas, resulting in variations in long-term services for older people.

Moreover, our results showed that the policies in the majority of pilot cities were designed to alleviate both the financial burdens and caregiving burdens faced by families, as well as to address the issue of “social hospitalization,” thereby improving the efficiency of the medical insurance. Recent research using quantitative methods also showed that LTCI in China had significantly reduced informal care intensity, family medical expenses and the length of hospital stays in pilot cities (14, 15, 18, 22). However, several challenges still remain.

First, we found that the average scores for all three indicators assessing LTCI’s responsiveness was below the overall average, indicating that, on average, the pilot programs did not adequately address the needs, preferences, and values of older people. In China, institutional care services have grown rapidly in recent years, whereas home and community-based care services remain underdeveloped (47). Over the past decade, initiatives have been launched in major cities to pilot various home and community-based care models, such as community stations, respite services, adult day centers, and home services. However, these measures were primarily concentrated in urban areas. Rural regions face obstacles due to resource limitations, insufficient policy focus, and the dispersed population. Moreover, the shortage of caregivers and inadequate quality of long-term care services also present challenges to the responsiveness of LTCI (48).

Second, inadequate coverage is another problem. Most of the pilot cities only covered urban employees and excluded rural residents. Based on our evaluation, Qingdao, was one of the pilot cities that demonstrated outstanding performance. However, the study by Zhu and Österle (31) showed that by the end of 2017, less than 2% of people aged 60 and over in Qingdao were benefiting from LTCI even though LTCI in Qingdao had relatively broader coverage compared to other cities. This suggests that the proportion of older people receiving LTCI benefits in other pilot cities, which exclusively covered urban employees, was even lower. This places LTCI program significant behind neighboring countries in terms of coverage. For instance, In Japan, 16.3% of individuals over the age of 65 were eligible for LTCI in 2005, with 13% actually utilizing services (41, 49, 50). In South Korea, the coverage ratio for older people was 8.8% in 2018 (51).

Finally, there were also other challenges, such as fragmentation of disability assessment standards, limited financing options, and an over-reliance on the social health insurance fund. These challenges have the potential to threaten the sustainability of LTCI. Addressing these issues is critical for the development of a robust long-term care regulatory framework (52).

This study has some potential limitations. A general drawback of qualitative approach is that the assessment may not be as objective as the quantitative data suggest, particularly in terms of the subjectivity of the scores assigned to each evaluation criterion. To mitigate the problem, we use the guidelines issued by China’s central government as a reference when assigning the scores. Furthermore, given that the pilot projects were initiated relatively recently, in 2016 and subsequently expanded in 2020, and that there is a typically time lag effect of the policies on the target population, our ability to conduct quantitative analysis is thus constrained. As more data become available, such as longitudinal family data, this could serve as a direction for future research aimed at investigating the effects of LTCI policies on the families.

Depending on the current status of LTCI in pilot cities, we propose several policy recommendations. First, authorities could consider separating the LTCI system from social health insurance system to establish an independent and self-sustaining LTCI. This can be achieved by clearly defining the relationship between medical insurance and long-term care insurance, effectively delineating the scope of treatments and payments between the two, and emphasizing the independence of LTCI in terms of its design and funding. For instance, for urban employees, a funding channel of “individual contributions + employer contributions + government contributions” could be employed, while for urban non-working residents and rural residents, a funding channel like “individual contributions + government contributions+ charity funds (e.g., public welfare lottery)” could be adopted. In addition, the funding methods could be adjusted from a fixed ratio to a variable ratio, allowing for dynamic adjustments in funding levels based on the local economic development level.

Second, when the pilot cities implement the LTCI programs, special attention should be given to the value dimension, with the goal of establishing an inclusive LTCI systems that grants an equal access for urban and rural residents as soon as possible. To achieve this, governments could prioritize the allocation of resources for the development of long-term care facilities in rural areas and consider providing subsidies to families with older people that are most in need, particularly those in less developed rural regions. The government can also provide some financial incentives, such as tax exemptions or subsidies, to private health care providers to encourage them to build care facilities in rural areas.

Third, regarding the issue of responsiveness in long-term care services, the government should provide professional training programs to improve the quality of care provided by long-term care workers. In addition, there is a need to support informal family caregivers by equipping them with the necessary caregiving knowledge and skills to become more efficient care providers. Policies should also focus on promoting community involvement and encouraging volunteerism, especially among youth and retired people. Offering flexible employment options, such as part-time work and adaptable working hours, to attract more people to work in long-term care services. Furthermore, integrating new technologies, such as AI technology, to enable more efficient care management and reduce the physical burden on caregivers.

Finally, in order to prevent or delay the onset of dementia, the governments could offer rehabilitative training and guidance support to individuals with mild to moderate disabilities and cognitive impairments.

## 5 Conclusion

In this paper, we present a comprehensive evaluation framework for assessing the LTCI policies by using qualitative data collected from the official documents of 49 pilot cities. The main conclusion of this paper is that the LTCI pilot program in China has achieved some positive outcomes, but also faces some challenges and variations across the pilot cities. Therefore, it is recommended that the government should improve the coordination and integration of the LTCI policies and services, and increase the availability and quality of the data and information for the evaluation and improvement of the LTCI system. It is worth noting that due to the relatively recent initiation of pilot projects, which were launched in 2016 and their subsequently expanded in 2020, the availability of micro-level data is limited. As a result, our evaluation is constrained to qualitative analysis. Future research could enhance the analysis by using longitudinal micro-level data, such as family panel data, to investigate the precise impact of LTCI policies on older people and their family members.

## Data availability statement

The original contributions presented in the study are included in the article/Supplementary material, further inquiries can be directed to the corresponding author.

## Author contributions

QL and YC: conceptualization and writing-review and editing. QL, YZ, and XL: data collection and methodology. QL: writing-original draft. All authors contributed to the article and approved the submitted version.

## Footnotes

^1^CHARLS stands for the China Health and Retirement Longitudinal Study while CLHLS is an abbreviation for the Chinese Longitudinal Healthy Longevity Study.

^2^Our assessment was based on the policy documents published up to 2021. A list of policy documents is presented in Appendix 1.

^3^The LTCI in all cities offers at least one type of long-term care services, being either daily life care or medical care.

^4^For all indicators, if not explicitly stated, a score of 0 was assigned to the cities, indicating that the policies or services are not explicitly mentioned in the official documents of the respective cities.

^5^In some cities like Shanghai and Suzhou, the LTCI is sorely financed by the existing social health insurance, specifically the Urban Employee Basic Medical Insurance (UEBMI) for urban employees and the Urban–Rural Resident Basic Medical Insurance (URRBMI) for rural residents and urban non-working residents. In some other cities, the LTCI is funded through a combination of sources including social health insurance, government contributions, individual contributions, employer contributions, and other funding channels.
