# Supplementary material for: Evaluation of China’s long-term care insurance policies
Source: Front Public Health. 2024 Mar 28;12:1252817. doi: 10.3389/fpubh.2024.1252817 (PMC11007106; doi:10.3389/fpubh.2024.1252817)
Supplement: Supplementary file 1 [file Data_Sheet_1.docx]

Supplementary Material

Evaluation of China’s long-term care insurance policies

**Qiang Li, Yiwen Chen^*,^ Yongmei Zhang, Xue Liu**

*** Correspondence:** Yiwen Chen: yiwen.chen@sdau.edu.cn

# Supplementary data

**Appendix 1**

**List of government policy documents**

Chengde Municipal Government. Implementation Opinions on the Establishment of Long-term Care Insurance System for Urban Employees (for Trial Implementation) [2016].

Changchun Municipal Government. Opinions on the Establishment of Medical Care Insurance System for Disabled people [2015].

Jilin Municipal Government.Opinions on the Implementation of Long-term Care insurance System (for Trial Implementation) [2016].

Tonghua Municipal Government. Implementation Opinions of the Office of the People's Government on the Pilot Project of Long-term Care Insurance System in Tonghua. [2017].

Songyuan Municipal Government. Opinions of the People's Government on the Pilot Project of Long-term Care Insurance System in Songyuan [2017].

Mehekou Municipal Government. Implementation Plans for the Pilot Project of Long-term Care Insurance System in Mehekou [2021].

Hunchun Municipal Government. Implementation Plans to Further Promote the Pilot Project of Long-term Care Insurance System in Hunchun (for Trial Implementation) [2021].

Qiqihar Municipal Government. Implementation Plans for Long-term Care Insurance in Qiqihar (for Trial Implementation) [2017]. Implementation Plan for Deepening Long-term Care Insurance System in Qiqihar (for Trial Implementation) [2021].

Shanghai Municipal Government. Pilot Scheme of Long-Term Care Insurance in Shanghai [2017]. Pilot Scheme of Long-Term Care Insurance in Shanghai (Revised) [2017]. Pilot Scheme of Long-term Care Insurance in Shanghai [2021].

Nantong Municipal Government. Opinions on the Establishment of Basic Care Insurance System (for Trial Implementation) [2015].

Suzhou Municipal Government. Opinions on the Implementation of Pilot Project of Long-term Care Insurance [2017]. Opinions on the Implementation of Pilot Project of the Second Phase of the Long-term Care Insurance [2021].

Ningbo Municipal Government. Pilot Project of Long-term Care Insurance System in Ningbo [2017].

Anqing Municipal Government. Implementation Opinions on the Pilot Project of Long-term Care Insurance for Urban Employees in Anqing [2017]. Implementation Measures for Long-term Care Insurance for Urban Employees in Anqing [2021].

Shangrao Municipal Government. Implementation Plans for Pilot Project of Long-term Care Insurance [2016]. Implementation Plans for Comprehensive Long-term Care Insurance System [2019].

Jingmen Municipal Government. Long-term Care Insurance Scheme in Jingmen (for Trial Implementation) [2016].

Guangzhou Municipal Government. Trial Measures for Long-term Care Insurance in Guangzhou [2017]. Trial Measures for Long-term Care Insurance in Guangzhou (Revised) [2019]. Trial Measures for Long-term Care Insurance in Guangzhou [2019].

Chongqing Municipal Government. Opinions on the Pilot Project of Long-term Care Insurance System in Chongqing [2017]. Opinions on the Implementation of the Expansion of Pilot Long-term Care Insurance System [2021].

Chengdu Municipal Government. Pilot Project for Long-term Care Insurance System in Chengdu [2017].

Shihezi Municipal Government. Opinions on the Establishment of Long-term Care Insurance System (for Trial Implementation) [2017].

Jinan Municipal Government. Opinions on the Establishment of a Long-term Medical Care Insurance System for Employees [2016]. Programme on the Expansion of the Pilot Long-term Care Insurance System in Jinan [2021].

Qingdao Municipal Government. Opinions on the Establishment of Long-term Medical Care Insurance System (for Trial Implementation) [2012]. Interim Management Measures of Long-Term Medical Care Insurance System [2015]. Interim Measures for Long-term Care Insurance in Qingdao [2018].

Zibo Municipal Government. Interim Measures for Long-term Care Insurance for Employees in Zibo (No.126 [2017]). Interim Measures for Long-term Care Insurance for Employees in Zibo (No.102 [2019]). Interim Measures for Long-term Care Insurance for Employees in Zibo (No.102 [2021]).

Zaozhuang Municipal Government. Opinions of the Office of the People's Government on the Establishment of a Long-term Care Insurance System for Employees in Zaozhuang [2018].

Dongying Municipal Government. Implementation Plans for the Pilot Project of Long-term Care Insurance for Urban and Rural Residents in Dongying [2018]. Implementation Plans for Improving the Long-term Care Insurance System [2021].

Yantai Municipal Government. Opinions of the Office of the People's Government on the Development of Long-term Care Insurance for Employees in Yantai [2018].

Weifang Municipal Government. Implementation Opinions on the Pilot Project of

Long-term Care Insurance for Employees [2014]. Implementation Plans on Long-term Care Insurance System for Urban and Rural Residents [2021].

Jining Municipal Government. Implementation Measures of Long-term Care Insurance for Employees in Jining [2018].

Tai'an Municipal Government. Notice on Trial Implementation of Long-term Care Insurance System for Employees in Tai’an [2017]. Notice on Issues Related to Improving the Long-term Care Insurance System for Employees in Taian [2021].

Weihai Municipal Government. Regulations on Long-term Care Insurance for Employees in Weihai [2018]. Notice on the Pilot Project of Long-term Care Insurance for Residents [2020].

Rizhao Municipal Government. Opinions of the Office of the Rizhao Municipal People's Government on Improving the Long-term Care Insurance System [2017]. The Implementation Opinions of the Office of the Rizhao Municipal People's Government on the Pilot Project of Long-term Care Insurance for Residents [2020].

Linyi Municipal Government. Notice of the Office of Municipal People's Government on the Issuance of the Implementation Plans of Long-term Care Insurance System for Employees in Linyi [2019].

Liaocheng Municipal Government. Opinions of Municipal People's Government Office on the Development of Long-term Care Insurance System for Employees in Liaocheng [2017].

Binzhou Municipal Government. Implementation Opinions of the Office of the People's Government on the Pilot Project of the Long-term Care Insurance System for Employees in Binzhou [2017].

Heze Municipal Government. Implementation Measures of Long-term Care Insurance for Employees in Heze [2021].

Beijing (Shijingshan District). The Pilot Project of Long-term Care Insurance System (for Trial Implementation) in Shijingshan District [2018].

Tianjing Municipal Government. Implementation Plans for the Pilot Project of Long-term Care Insurance System in Tianjin [2020].

Jincheng Municipal Government. Implementation Opinions of the People's Government on the Establishment of Long-term Care Insurance System in Jincheng [2020].

Hohhot Municipal Government. Implementation Plans for the Pilot Project of Long-term Care Insurance System in Hohhot [2020].

Panjin Municipal Government. Implementation Plans for Pilot Project of Long-term Care Insurance System in Panjin [2020].

Fuzhou Municipal Government. Notice of Municipal People's Government on the Implementation Plans for the Pilot Project of Long-term Care Insurance System in Fuzhou [2020].

Kaifeng Municipal Government. Trial Measures of Long-term Care Insurance System in Kaifeng [2021].

Xiangtan Municipal Government. Implementation Plans for the Pilot Project Long-term Care Insurance System Pilot in Xiangtan [2020].

Nanning Municipal Governments. Opinions on the Implementation of the Pilot Project of Long-term Care Insurance System in Nanning [2021].

Kunming Municipal Governments. Implementation Plans of People's Government on the Comprehensive Launch of the Pilot Project of Long-term Care Insurance System in Kunming [2020].

Hanzhong Municipal Governments. Implementation Measures for Long-term Care Insurance in Hanzhong (for Trial Implementation) [2020].

Gannan Tibetan Autonomous Prefecture. Rules for the Implementation of Long-term Care Insurance for Employees in Gannan (for Trial Implementation) [2021].

Urumqi Municipal Governments. Urumqi Long-term Care Insurance Measures (for Trial Implementation) [2019].

**Appendix 2**

**Funding sources of long-term care insurance (LTCI) in China**

Due to the significant disparity in the financing sources of long-term care insurance (LTCI) between urban employees and urban non-working and rural residents, we illustrate the funding channels separately.

*The funding sources of long-term care insurance (LTCI) for urban employees*

The funding sources of LTCI programs for urban employees are mainly divided into two types: LTCI in cities like Shanghai and Suzhou, is financed by a single channel, that is, long-term care insurance funds solely come from the basic social health insurance funds UEBMI; The other type is the multi-financing channels, which can be further divided into two categories. The first category is the multi-funding channel primarily based on UEBMI, and the other one is the multi-funding channels primarily based on “employer contributions + individual contributions”. Specifically, the multi-funding channels that are primarily based on UEBMI include six modes. The first is the “UEBMI + individual contributions” represented by cities like Chongqing, Jilin and Tonghua; the second mode is the “UEBMI + individual contributions + government contributions” represented by cities like Chengde and Nantong; the third mode is the “UEBMI + individual contributions + government contributions + public welfare lottery” represented by cities like Yantai and Jining; the fourth one is the “UEBMI + individual contributions + employers contributions + public welfare lottery” represented by cities like Liaocheng; the fifth one is the “UEBMI + government contributions + public welfare lottery” represented by cities like Shihezi and Jinan; the sixth mode is the “UEBMI + individual contributions + employer contributions” represented by cities like Shangrao. The multi-funding channels that are primarily based on “employer contributions + individual contributions” include three modes. The first is the “employer contributions+ individual contributions”, which has been adopted by many pilot cities, such as Changchun, Qiqihar and Ningbo; the second mode is the “employer contributions + individual contributions + government contributions” represented by cities like Chengdu, Jincheng, etc.; the third one is the “employer contributions + individual contributions+ government contributions + public welfare lottery” represented by cities Qianxinan and Binzhou.

*The funding sources of long-term care insurance (LTCI) for urban non-working residents and rural residents*

The financing sources of LTCI for urban non-working residents and rural residents can be divided into six modes: the first mode is that LTCI is solely financed URRBMI represented by cities like Shanghai and Suzhou; the second mode is the “URRBMI + government subsidies” represented by cities like Yantai and Rizhao; the third one is “URRBMI + government subsidies + individual contributions” represented by cities like Nantong, Shangrao and Jingmen; the fourth mode is “URRBMI + government subsidies+ public welfare lottery + individual contributions” represented by cities like Weihai; the fifth is the “individual contributions + government subsidies”, which is adopted by pilot cities, such as Changchun, Qingdao and Guangzhou, etc.; the sixth mode is the “individual contributions + government subsidies + public welfare lotteries” represented by cities like Shihezi.

**Appendix 3**

**Assessment scores of LTCI policies in each pilot city**

| **Performance** | **Pilot**  **cities** | **Fact-based assessment** | **Value-based assessment** | **Total Score** |
| --- | --- | --- | --- | --- |
| Excellent | Qingdao | 43.0 | 49.5 | 92.5 |
|  | Jingmen | 38.5 | 49.0 | 87.5 |
|  | Shanghai | 43.0 | 39.0 | 82.0 |
|  | Nantong | 36.5 | 44.5 | 81.0 |
|  | Chengdu | 34.0 | 46.5 | 80.5 |
|  | Suzhou | 40.0 | 40.0 | 80.0 |
|  | Guangzhou | 38.5 | 41.5 | 80.0 |
|  | Hohhot | 36.5 | 43.5 | 80.0 |
|  | Jinan | 40.0 | 40.0 | 80.0 |
| Good | Shangrao | 36.0 | 41.0 | 77.0 |
|  | Zibo | 36.0 | 38.5 | 74.5 |
|  | Linyi | 37.5 | 36.0 | 73.5 |
|  | Yantai | 31.0 | 42.0 | 73.0 |
|  | Kunming | 33.5 | 39.0 | 72.5 |
|  | Kaifeng | 35.0 | 37.5 | 72.5 |
|  | Binzhou | 36.0 | 36.5 | 72.5 |
|  | Changchun | 29.5 | 43.0 | 72.5 |
|  | Shijingshan | 32.5 | 40.0 | 72.5 |
|  | Hanzhong | 32.5 | 40.0 | 72.5 |
|  | Rizhao | 32.5 | 40.0 | 72.5 |
|  | Dongying | 33.5 | 38.5 | 72.0 |
|  | Anqing | 34.0 | 37.5 | 71.5 |
|  | Jining | 34.5 | 37.0 | 71.5 |
|  | Taian | 35.5 | 36.0 | 71.5 |
|  | Heze | 37.0 | 34.5 | 71.5 |
|  | Shihezi | 31.5 | 40.0 | 71.5 |
|  | Urumqi | 33.0 | 38.5 | 71.5 |
|  | Chengde | 33.5 | 37.5 | 71.0 |
|  | Weifang | 36.0 | 35.0 | 71.0 |
|  | Xiangtan | 34.0 | 36.5 | 70.5 |
|  | Zaozhuang | 35.0 | 35.0 | 70.0 |
|  | Qianxinan Autonomous Prefecture | 32.5 | 37.5 | 70.0 |
| Fair | Jincheng | 33.0 | 36.5 | 69.5 |
|  | Nanning | 32.5 | 37.0 | 69.5 |
|  | Tianjin | 33.5 | 35.5 | 69.0 |
|  | Dezhou | 34.5 | 34.5 | 69.0 |
|  | Mehekou | 26.0 | 43.0 | 69.0 |
|  | Songwon | 27.0 | 41.5 | 68.5 |
|  | Fuzhou | 32.5 | 36.0 | 68.5 |
|  | Weihai | 28.0 | 40.0 | 68.0 |
|  | Liaocheng | 31.0 | 34.5 | 65.5 |
|  | Huichun | 26.0 | 39.0 | 65.0 |
|  | Chongqing | 32.5 | 32.5 | 65.0 |
|  | Gannan | 30.0 | 34.5 | 64.5 |
|  | Jilin | 27.0 | 37.5 | 64.5 |
|  | Tonghua | 26.0 | 36.5 | 62.5 |
|  | Panjin | 28.5 | 32.5 | 61.0 |
|  | Qiqihar | 28.5 | 32.0 | 60.5 |
|  | Ningbo | 25.5 | 32.0 | 57.5 |
|  | Average value | 33.35 | 38.48 | 71.83 |

**Appendix 4**

| Tier 1  Dimension | Tier 2  Subdimensions | Tier 3  Indicators | Scoring Criteria | | | |
| --- | --- | --- | --- | --- | --- | --- |
|  |  |  | 5 Points | 4 Points | 2.5 Points | 0 Points |
| Fact | Effectiveness | Direct  effects | Provision of long-term care services, that is, the service covering both basic life care services such as diet care and excretion care, as well as long-term medical care services such as nasogastric tube placement and bladder irrigation. | Mainly focusing on long-term medical care services or long-term life care services. | Only several service items in long-term medical care services or long-term life care services are provided. | No long-term medical care or long-term life care services are provided |
|  |  | Indirect effects | The level of long-term care insurance benefits (reimbursement ratio) is higher than 75 %. | The level of benefit payment (reimbursement ratio) is between 65 % and 75 %. | The level of benefit payment (reimbursement ratio) is less than 65 %. | No reimbursement is provided. |
|  |  | Potential effects | It provides all three levels of services: specialized nursing care, institutional care and home care. | It provides two of the three levels of services. | Only one of the three levels of services is provided. | No medical care services are provided. |
|  | Impact | Care services for the aged | It provides both institutional care and home care. | It provides both institutional care and home care, but the segmentation of the two types of services is partially lacking. | It provides only one type of care, being either institutional care or home care. | Neither of care is provided. |
|  |  | Medical payment coverage | It covers not only long-term care services, equipment use and consumables, but also treatment, drugs and bed fees | It covers the long-term care services, equipment uses, and related consumables. | It covers only the long-term care services | It covers less than the long-term care service fees and only contains very few items. |
|  |  | Long-term care service industry | The pilot cities have introduced a comprehensive set of incentive policies to fully support the development of long-term care institutions and implemented talent training programs. | The pilot cities have provided partial support for long-term care institutions and talent training programs. | The pilot cities offer limited support to the long-term care service industry and lack policies for relevant professional trainings. | The pilot cities provide little support to the long-term care service industry. |
|  | Responsiveness | Group-specific needs | The pilot cities that have comprehensively addressed the needs of different disability levels. | The pilot cities provide treatment for the severe and some moderate disability groups | The pilot cities only offer treatment for the severe disabled and have insufficient response to the needs. | The pilot cities don’t provide treatment for people with different degrees of disability. |
|  |  | Group-specific preferences | The pilot cities offer life-cycle services and specifically focus on providing hospice care and other services for terminally disabled individuals. | The pilot cities offer long-term care services throughout the life cycle, including medical care, but do not offer end-of-life care services for the terminally disabled. | The pilot cities solely offer long-term life care services or incomplete long-term medical care services | The pilot cities only offer limited long-term life care services, and their response to the service preferences of disabled groups is extremely low. |
|  |  | Group-specific values | The pilot cities offer community care and different types of home care, demonstrating a commitment to the value of "aging in place" for the disabled group | The pilot cities offer basic home care and supplementary home care but being lack of community care. | The pilot cities offer only one type of care, being either at home or in the community | The pilot cities fail to provide home and community care services, and cannot meet the values of “aging in place”. |
| Value Evaluation | Equity | Equity in defining the target population | LTCI in the pilot cities cover both urban employees and urban and rural residents, extending the coverage from the physically disabled group to the mentally disabled group. | LTCI in the pilot cities cover urban employees and urban and rural residents, but the coverage was limited to the physically disabled group. | LTCI in the pilot cities cover only physically disabled group of urban employees. | LTCI in the pilot cities don’t cover the disabled groups. |
|  |  | Equity in outcomes | The pilot cities assess the disability level based on the ability to perform activities of daily living, cognitive ability, and perceptual ability ensure equal treatment outcomes. | The pilot cities use the ability to perform activities of daily living and the degree of illness as the criteria for determining disability. | The pilot cities define the level of disability based solely on the ability to perform daily living activities | The criteria for determining the level of disability are not clear. |
|  |  | Equity in rights | The pilot cities impose no restrictions on identity status and age for participating in LTCI. | The pilot cities have certain restrictions on the age and identity status of urban and rural residents. | The pilot cities only provide insurance coverage for urban employees and exclude urban and rural residents | Residents are unable to participate in LTCI. |
|  |  | Equity in opportunities | The pilot cities impose no stringent conditions, and only take the level of disability of people as eligibility criteria. | Except for taking the level of disability of people as eligibility criteria, the pilot cities impose one additional condition. | Except for taking the level of disability of people as eligibility criteria, the pilot cities impose two additional conditions. | Except for taking the level of disability of people as eligibility criteria, the pilot cities impose more than two additional conditions. |
|  | Sustainability | Financial sustainability | The pilot cities use multiple funding channels, dynamically adjustable standards, and a relatively low proportion of medical insurance funding. | The pilot cities use diversified funding channels, dynamically adjustable standards, but a high proportion of medical insurance funding | The pilot cities use dynamically adjustable funding levels but lack diversification in funding sources | The financing channel is not dynamically adjusted, and is not sustainable. |
|  |  | Sustainability of service delivery | The pilot cities offer a diverse range of services, including hospital care, nursing home care, full-day home care, and part-day home care. | The pilot cities provide multiple types of services but lack sufficient flexibility in transferring between them. | The pilot cities solely offer a single type of service. | No such type of services is provided. |
|  |  | Sustainability of administrative operations | The pilot cities actively engage commercial insurance companies in administrative operations and establish comprehensive performance evaluation and incentive mechanisms to improve the efficiency of management and services. | The pilot cities involve commercial insurance companies in administrative operations but lack a comprehensive assessment mechanism. | Commercial insurance companies have not been introduced to participate in the operation of the organization, but performance evaluation, assessment and incentives, and policies and norms are relatively perfect. | The pilot cities fail to introduce the participation of social forces in the operation, and many deficiencies in the assessment norms and other aspects of the operation of the organization. |
|  |  | Sustainability of policy coherence | The pilot cities exhibit clear scope of reimbursement and robust coordination among relevant departments. The government department that is in charge of LTCI has strong connections with other social insurance programs like medical insurance and work injury insurance. | The pilot cities exhibit good policy coherence. The government department that is in charge of LTCI has good connections with other social insurance programs. | The government department that is in charge of LTCI in the pilot cities has some connections with rother social insurance programs like medical insurance and work injury. But there exist some coordination problems among them, such as the lack of inefficiency. | The local government in that city is one of the main funding bodies or provides subsidies solely for the individual contributions of vulnerable groups. |
|  | Productivity | Inclusiveness | The pilot cities have the local authority as the main funding body and provide full subsidies for the individual contributions of vulnerable groups. | The local government in that city is a major funding body and partially subsidizes the individual contributions of vulnerable groups. | The local government in that city is one of the main funding bodies or provides subsidies solely for the individual contributions of vulnerable groups | The local government in that city is not one of the main funding bodies or doesn’t provide any subsidies for the individual contributions of vulnerable groups. |
|  |  | Adequacy | The funding level of LTCI in the pilot cities fully aligns with the level of economic development. | The funding level of LTCI in the pilot cities partially corresponds to the economic development level. | The funding level of LTCI in the pilot cities is slightly below their economic development level. | The funding level of LTCI in the pilot cities is far lagged behind their economic development level. |
|  |  | Scientificity | The pilot cities actively promote the construction of system platforms and successfully implement intelligent operational processes and scientific service supervision within their long-term care insurance systems. | The pilot cities actively promote the construction of system platforms, building a basic information archive of disabled persons, and adopting intelligent methods, but there are deficiencies in scientific service supervision within their long-term care insurance systems. | The pilot cities focus on the construction of the system platform, but there are deficiencies in implementation of intelligent operational processes and the scientific service supervision within their long-term care insurance systems. | The pilot cities have been slow in the construction of system platforms and are not able to implement intelligent operational processes and scientific service supervision within their long-term care insurance systems. |
